# The Comparison of the Effectiveness of Dapagliflozin and Empagliflozin in the Prevention of Cardiovascular Outcomes in Patients With Type 2 Diabetes: A Network Meta-Analysis

**DOI:** 10.7759/cureus.69711

**Published:** 2024-09-19

**Authors:** Tanya Sinha, Ushna Gul, Nawabzada Nadir Babar, Farhan Israr, Aqsa A Butt, Sandipkumar S Chaudhari, Hamza Maqbool, Adil Amin

**Affiliations:** 1 Internal Medicine, Tribhuvan University, Kathmandu, NPL; 2 Internal Medicine, Khyber Medical College, Peshawar, PAK; 3 Internal Medicine, Combined Military Hospital (CMH) Lahore Medical College and Institute of Dentistry, Lahore, PAK; 4 Medicine, Khyber Medical College, Peshawar, PAK; 5 Internal Medicine, Lady Reading Hospital, Peshawar, PAK; 6 Internal Medicine, Allama Iqbal Medical College, Lahore, PAK; 7 Cardiothoracic Surgery, University of Alabama at Birmingham, Birmingham, USA; 8 Family Medicine, University of North Dakota School of Medicine and Health Sciences, Fargo, USA; 9 Internal Medicine, Continental Medical College, Lahore, PAK; 10 Cardiology, Pakistan Navy Ship (PNS) Shifa, Karachi, PAK

**Keywords:** cardiovascular outcomes, dapagliflozin, empagliflozin, systematic review and meta-analysis, type 2 diabetes mellitus

## Abstract

Type 2 diabetes mellitus (T2DM) is a significant risk factor for cardiovascular diseases, prompting research into treatments that can mitigate this risk. Sodium-glucose cotransporter 2 (SGLT2) inhibitors, particularly dapagliflozin and empagliflozin, have shown promising cardiovascular benefits in T2DM patients. This meta-analysis aimed to directly compare the cardiovascular outcomes of these two drugs. To achieve this, we conducted a comprehensive literature search across multiple databases up to August 5, 2024, including both randomized controlled trials (RCTs) and observational studies. The primary outcomes of interest were major adverse cardiovascular events (MACE), atrial fibrillation (AF), cardiovascular mortality, myocardial infarction (MI), and hospitalization for heart failure (HF). Twelve studies met the inclusion criteria for this meta-analysis. The pooled analysis revealed several key findings. Notably, dapagliflozin demonstrated superior efficacy in preventing atrial fibrillation compared to empagliflozin. However, no significant differences were observed between the two drugs in terms of MACE, cardiovascular mortality, hospitalization for heart failure (HHF), or myocardial infarction. When compared to placebo, both dapagliflozin and empagliflozin showed greater effectiveness in preventing adverse cardiovascular outcomes in T2DM patients. These results reinforce the cardiovascular benefits of both dapagliflozin and empagliflozin in patients with T2DM. The comparable efficacy in most outcomes suggests that clinicians have flexibility in prescribing either of these SGLT2 inhibitors. However, the lower risk of atrial fibrillation associated with dapagliflozin may be a crucial factor in treatment decisions, especially for patients with a history of or at high risk for atrial fibrillation.

## Introduction and background

Type 2 diabetes mellitus (T2DM) is a widespread global health crisis, with projections estimating that 640 million people will be affected by 2040 [1]. T2DM is closely linked to an increased risk of cardiovascular ischemic events and hospitalizations for heart failure (HHF), independent of other factors [2,3]. Despite current standard treatments, the residual risk in T2DM patients remains significantly high so much so that these patients have shown substantial benefits in reducing major adverse cardiovascular events (MACE) through advanced secondary prevention strategies, such as prolonged dual antiplatelet therapy [4] and more intensive lipid-lowering treatments [5] following a myocardial infarction (MI). Furthermore, individuals with a history of MI are at a greater risk of developing heart failure (HF) and face reduced long-term survival rates [6].

To reduce these risks, blood glucose control must be done well. In this regard, sodium-glucose cotransporter 2 (SGLT2) inhibitors such as dapagliflozin and empagliflozin have become well-known medications. Compared to conventional glucose-lowering medications, these inhibitors have a number of benefits, such as the ability to lower blood pressure and promote weight loss without raising the risk of hypoglycemia [7,8]. In the field of managing diabetes, dapagliflozin and empagliflozin are unique due to their proven benefits for the cardiovascular system. Their effectiveness in lowering cardiovascular risks in patients with diabetes mellitus (DM) and heart failure with a decreased ejection fraction has been demonstrated in multiple clinical trials [9,10]. Recent studies have demonstrated that their benefits also extend to patients with heart failure with preserved ejection fraction [11]. In response to these findings, current guidelines now recommend the use of SGLT2 inhibitors for heart failure patients, regardless of their ejection fraction status [12].

Notwithstanding these developments, there is still a significant lack of real-world data, especially when it comes to a direct comparison of dapagliflozin with empagliflozin in DM patients. This is particularly true in international settings where prescriptions for these drugs are common. Closing this gap is critical to improving patient outcomes and making well-informed healthcare decisions. In order to compare the efficacy of empagliflozin with dapagliflozin in avoiding atrial fibrillation (AF) and adverse cardiovascular events in individuals with type 2 diabetes (T2D), we are performing this meta-analysis.

## Review

Methodology

Following the Preferred Reporting Items for Systematic Reviews and Meta-Analyses (PRISMA) criteria, this meta-analysis was carried out and reported. We searched for databases including Scopus, Embase, PubMed, and Web of Science to search for all relevant studies published from inception to August 5, 2024. Keywords used to search for studies include "empagliflozin," "dapagliflozin," "type 2 diabetes," and "cardiovascular outcomes." These keywords were combined along with Medical Subject Heading (MeSH) terms using Boolean algebra operators. The manual screening of the reference list of included trials was used to identify any related studies that may have been missed during the search. The search was performed by two authors independently. Any disagreement between the two authors was resolved through consensus.

Study Selection and Eligibility Criteria

We included studies that met the following inclusion criteria: (1) included adult patients (age of >18 years); (2) patients with T2DM with and without heart failure; (3) used drugs were dapagliflozin, empagliflozin, and placebo; (4) randomized controlled trials (RCTs) or observational studies; and (5) studies that reported at least one of the outcomes assessed in this meta-analysis.

All records exported from target databases were first imported into the EndNote software (Clarivate, Philadelphia, PA). Duplicate records were then removed by running the function of locating duplicate items embedded in the EndNote software. In the second step, the titles and abstracts of unique records were screened for initial eligibility evaluation, and potentially eligible records were stored in a separate file for further evaluation. In the third step, we accessed the full texts of all potentially eligible records and then tested for eligibility by screening the full texts. Study selection was performed by two authors. Any disagreement was resolved through consensus.

Quality Assessment

The quality of the included studies was assessed using specific tools tailored to their design. For randomized controlled trials (RCTs), we employed the Cochrane Risk of Bias Assessment Tool (Cochrane, London, England), which evaluates bias across several domains, including selection, performance, detection, attrition, and reporting bias. Each domain was rated as low, high, or unclear risk. For observational studies, we used the Newcastle-Ottawa scale (NOS), assessing study quality based on selection, comparability, and outcome/exposure ascertainment. The NOS provides a score reflecting the study's overall quality, with higher scores indicating a lower risk of bias. These assessments ensured a rigorous evaluation of study reliability.

Data Extraction and Outcome Measures

We designed a data extraction sheet based on Microsoft® Excel software (Microsoft Corp., Redmond, WA), which was used by two independent reviewers to extract the following information from each eligible study: the name of the first author, publication year, study design, comparison, sample size, the proportion of male patients, mean age, the time of follow-up, and outcomes. Outcomes assessed in this meta-analysis included major adverse cardiovascular events (MACE), the incidence of atrial fibrillation, cardiovascular mortality, myocardial infarction, and hospitalization for heart failure. Any disagreement regarding data extraction was resolved by consulting a third reviewer. For the network meta-analysis (NMA), the quality of direct and network evidence was graded using the Grading of Recommendations Assessment, Development and Evaluation (GRADE). Each comparison of the quality of direct and network evidence for each outcome was done.

Statistical Analysis

All analyses were conducted using RStudio (Posit PBC, Boston, MA). Risk ratios (RR) with 95% confidence intervals (95% CI) were calculated to assess binary variables. A fixed-effect model was applied if I^2^ < 50% and p > 0.01; otherwise, a random effects model was used. Network meta-analyses (NMA) were performed under the frequentist framework in the RStudio software using a random effects model. Matrices, expressed as RR and 95% CI, were created to display the pairwise comparisons of all interventions for each endpoint. To improve result stability, both overall and loop inconsistencies between direct and indirect comparisons were evaluated. Funnel plots were used to assess the small sample effect. A p-value of <0.05 was considered statistically significant.

Results

**Figure 1:**
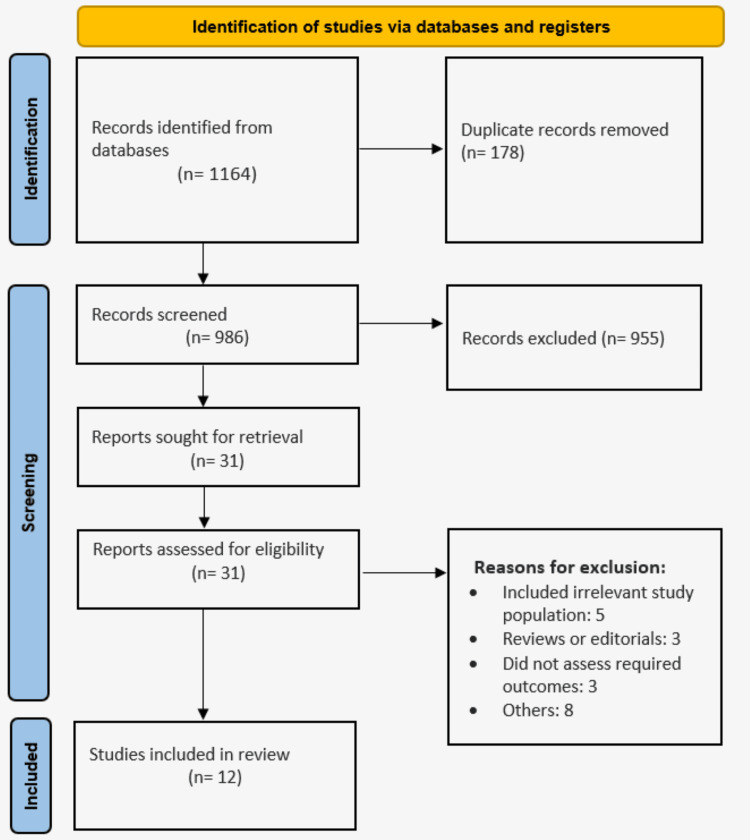
PRISMA flowchart of the study selection process PRISMA: Preferred Reporting Items for Systematic Reviews and Meta-Analyses

We acquired 1164 papers by incorporating the investigations conducted up until August 5, 2024. Twelve articles were included in this meta-analysis after 986 studies were screened and duplicates were removed, followed by a full-text screening process based on inclusion criteria. Figure 1 displays the flow chart for screening. Table 1 displays the primary attributes of all the included studies. Seven of the 12 studies were observational, while five of the studies were RCTs. Table 2 presents the risk of bias assessment of the included RCTs. Table 3 presents the quality assessment of observational studies. GRADE NMA assessment has been presented in the Appendices.

**Table 1:** Included study characteristics RCT, randomized controlled trial; NR, not reported

Author	Year	Design	Groups	Sample size	Follow-up	Dose of drug	Age (years)	Males (n)
Anker et al. [13]	2021	RCT	Empagliflozin	927	24 months	10 mg	66.8	717
			Placebo	929			66.6	710
Barnett et al. [14]	2014	RCT	Empagliflozin	195	12 months	10 or 25 mg	62.3	121
			Placebo	95			62.6	56
Kim et al. [15]	2024	Observational	Empagliflozin	537	40.9 months	NR	57.7	353
			Dapagliflozin	537			57	350
Lim et al. [16]	2023	Observational	Empagliflozin	72752	24.96 months	10 or 25 mg	56.1	42300
			Dapagliflozin	72752			55.8	42403
Lim et al. [17]	2024	Observational	Empagliflozin	68964	26.4 months	10 mg	55.8	39991
			Dapagliflozin	68964			55.4	40236
Ozbek and Can [18]	2023	Observational	Dapagliflozin	120	4 months	10 mg	64.81	62
			Placebo	326			64.14	151
Park et al. [19]	2022	Observational	Empagliflozin	600	39.9 months	NR	NR	NR
			Dapagliflozin	609			NR	NR
Petrie et al. [20]	2020	RCT	Dapagliflozin	1075	18.4 months	5 mg	66.3	835
			Placebo	1064			66.7	827
Shao et al. [21]	2019	Observational	Empagliflozin	5812	21.86 months	10 or 25 mg	NR	2892
			Dapagliflozin	6869			NR	4222
Wiviott et al. [22]	2019	RCT	Dapagliflozin	8582	50.4 months	10 mg	63.9	5411
			Placebo	8578			64	5327
Zelniker et al. [23]	2020	RCT	Dapagliflozin	8578	50.4 months	NR	NR	NR
			Placebo	8582			NR	NR
Zinman et al. [24]	2015	RCT	Empagliflozin	4687	48 months	10 or 25 mg	NR	NR
			Placebo	2333			NR	NR

**Table 2:** Risk of bias assessment of included studies (RCTs) RCTs: randomized controlled trials

Author ID	Random sequence generation	Allocation concealment	Blinding of participants	Blinding of outcome assessor	Incomplete outcome data	Selective reporting	Other bias
Anker et al., 2021 [13]	Low	Low	Low	Low	Low	Low	Low
Barnett et al., 2014 [14]	Low	Low	Low	Low	Low	Low	Low
Petrie et al., 2020 [20]	Low	Unclear	Low	Low	High	Low	Unclear
Wiviott et al., 2019 [22]	Low	Low	Low	Low	Unclear	Unclear	Low
Zelniker et al., 2020 [23]	Low	Low	Low	Low	Low	Low	Low
Zinman et al., 2015 [24]	Low	Low	Low	Low	Unclear	Low	Unclear

**Table 3:** Quality assessment of included studies (observational studies)

Study ID	Selection	Comparability	Assessment	Overall
Kim et al., 2024 [15]	4	2	3	Good
Lim et al., 2023 [16]	3	1	2	Fair
Lim et al., 2024 [17]	3	2	3	Good
Ozbek and Can, 2023 [18]	4	2	3	Good
Park et al., 2022 [19]	3	2	2	Good
Petrie et al., 2020 [20]	3	2	3	Good
Shao et al., 2019 [21]	3	1	3	Good

MACE 

**Figure 2:**
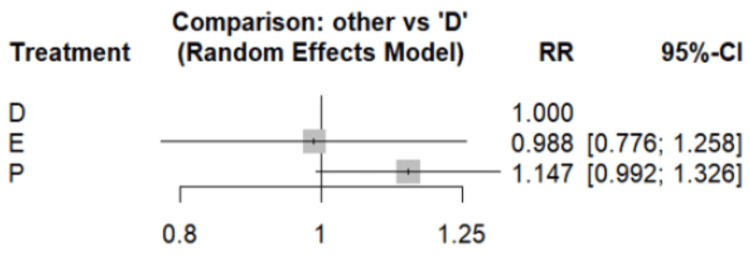
Forest plot comparing MACE References: [15,18,20,22,24] D, dapagliflozin; E, empagliflozin; P, placebo; RR, risk ratio; CI, confidence interval; MACE, major adverse cardiovascular events

Figure 2 presents the risk of developing MACE between dapagliflozin and empagliflozin. The analysis revealed no significant difference in MACE risk between dapagliflozin and empagliflozin, with a risk ratio of 0.988 (95% CI: 0.776-1.258). Network meta-analysis results, presented in Table 4, compared the risk of major adverse cardiovascular events (MACE) among three groups including dapagliflozin and empagliflozin and placebo. The pooled analysis showed that both empagliflozin and dapagliflozin groups demonstrated a lower risk of MACE compared to placebo. Table 5 displays the surface under the cumulative ranking (SUCRA) scores, which further support these findings. Both empagliflozin and dapagliflozin emerged as the most effective treatments for MACE prevention, achieving the highest SUCRA ratings.

**Table 4:** Risk of outcomes among different outcomes (pairwise comparison) The result presented as a risk ratio (95% confidence interval) MACE: major adverse cardiovascular events

Outcome	Intervention			
		Dapagliflozin	Empagliflozin	Placebo
MACE	Dapagliflozin	1	1.01 (0.80-1.29)	0.87 (0.75-1.01)
	Empagliflozin	0.99 (0.78-1.26)	1	0.86 (0.70-1.06)
	Placebo	1.15 (0.99-1.33)	1.16 (0.95-1.42)	1
Atrial fibrillation	Dapagliflozin	1	0.89 (0.79-0.99)	0.81 (0.69-0.94)
	Empagliflozin	1.13 (1.01-1.26)	1	0.91 (0.75-1.10)
	Placebo	1.24 (1.06-1.45)	1.10 (0.91-1.34)	1
Cardiovascular mortality	Dapagliflozin	1	0.94 (0.65-1.34)	0.79 (0.57-1.09)
	Empagliflozin	1.07 (0.75-1.53)	1	0.84 (0.61-1.17)
	Placebo	1.27 (0.92-1.75)	1.18 (0.86-1.64)	1
Myocardial infarction	Dapagliflozin	1	1.02 (0.90-1.16)	0.89 (0.79-1.00)
	Empagliflozin	0.98 (0.86-1.11)	1	0.87 (0.75-1.01)
	Placebo	1.12 (1.00-1.26)	1.15 (0.99-1.33)	1
Hospitalization for heart failure	Dapagliflozin	1	0.98 (0.81-1.20)	0.70 (0.59-0.84)
	Empagliflozin	1.02 (0.84-1.23)	1	0.71 (0.59-0.86)
	Placebo	1.43 (1.20-1.73)	1.41 (1.17-1.69)	1

**Table 5:** SUCRA score of each drug SUCRA, surface under the cumulative ranking; MACE, major adverse cardiovascular events

Outcomes	Dapagliflozin	Empagliflozin	Placebo
MACE	0.665	0.79	0.045
Atrial fibrillation	0.985	0.445	0.07
Cardiovascular mortality	0.845	0.555	0.1
Myocaridal infarction	0.66	0.81	0.03
Hospitalization for heart failure	0.815	0.685	0.001

Atrial Fibrillation

**Figure 3:**
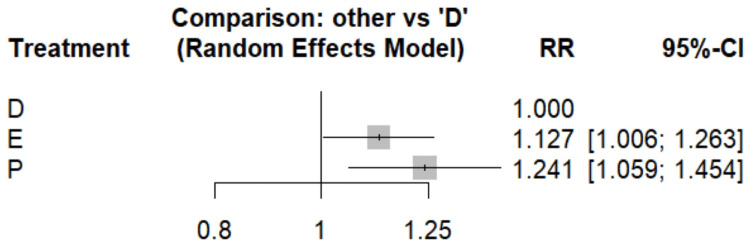
Forest plot comparing the risk of atrial fibrillation References: [14,17,19,23] D, dapagliflozin; E, empagliflozin; P, placebo; RR, risk ratio; CI, confidence interval

Figure 3 compares the risk of atrial fibrillation in dapagliflozin with empagliflozin. As compared with dapagliflozin, empagliflozin is associated with an increased risk of atrial fibrillation (RR, 1.127; 95% CI, 1.006-1.263). Table 4 showed the comparison of these drugs to placebo. Empagliflozin, when compared to placebo, showed a trend toward reduced atrial fibrillation risk with a risk ratio of 0.91 (95% CI: 0.75-1.10). However, this result did not reach statistical significance. The SUCRA scores further support these findings, with dapagliflozin achieving the highest score (0.985), followed by empagliflozin (0.445) and placebo (0.07). This ranking suggests that dapagliflozin may be the most effective treatment for preventing atrial fibrillation among the three options, followed by empagliflozin, with placebo being the least effective.

Cardiovascular Mortality

**Figure 4:**
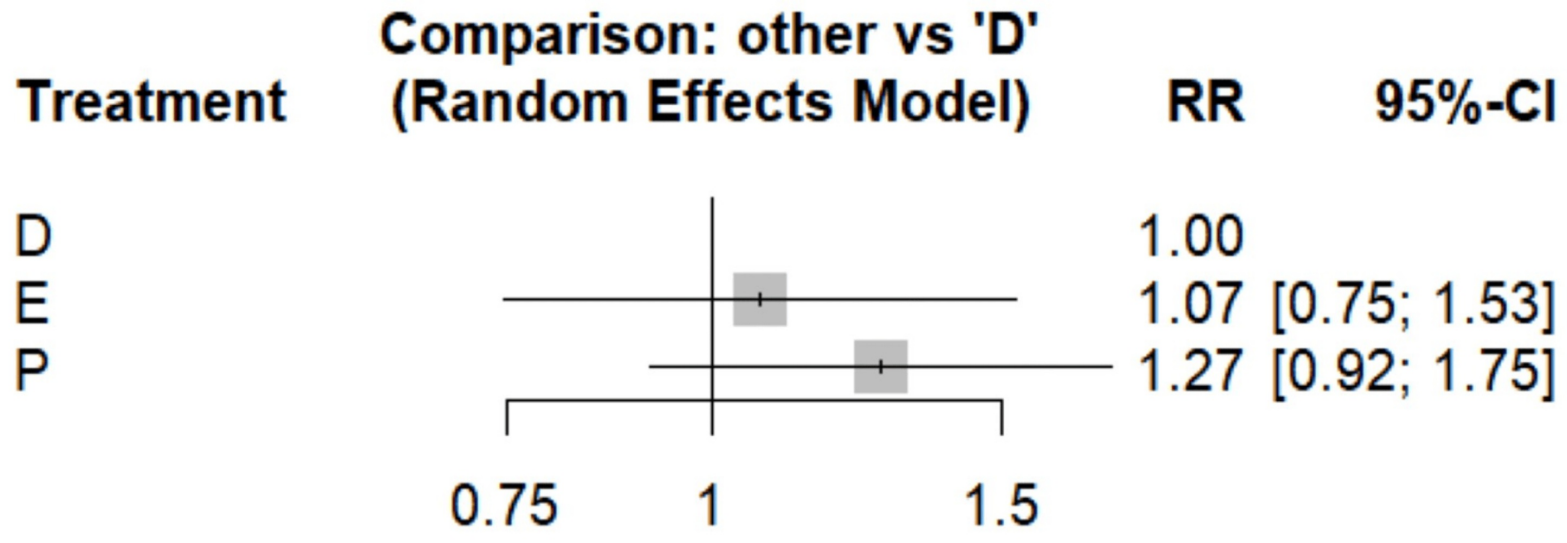
Forest plot comparing the risk of cardiovascular mortality References: [13,15,16,19,20,22,24] D, dapagliflozin; E, empagliflozin; P, placebo; RR, risk ratio; CI, confidence interval

Figure 4 assesses the risk of cardiovascular mortality between dapagliflozin and empagliflozin. The pooled analysis did not show any significant difference between two drugs. Dapagliflozin showed a 21% reduction (RR, 0.79; 95% CI, 0.57-1.09) and empagliflozin a 16% reduction (RR, 0.84; 95% CI, 0.61-1.17) compared to placebo as shown in Table 4. SUCRA scores (dapagliflozin, 0.845; empagliflozin, 0.555; and placebo, 0.1) indicate that dapagliflozin may be the most effective in reducing cardiovascular mortality risk, followed by empagliflozin, with placebo ranking last.

Myocardial Infarction 

**Figure 5:**
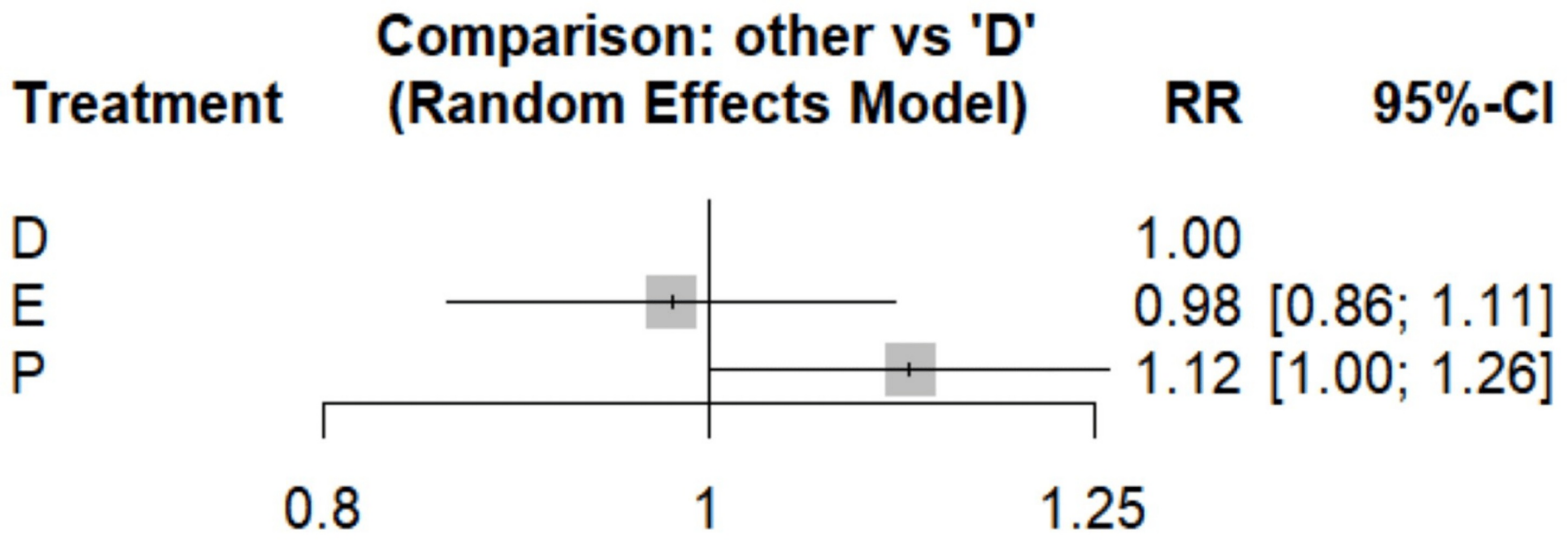
Forest plot comparing the risk of myocardial infarction References: [15,17,19,20,24] D, dapagliflozin; E, empagliflozin; P, placebo; RR, risk ratio; CI, confidence interval

Figure 5 compares the risk of myocardial infarction between dapagliflozin and empagliflozin. No significant difference was observed between the two groups indicating no clear superiority between these two agents. Table 4 showed a heat plot comparing these two drugs to placebo. Empagliflozin emerges as a potential front-runner, demonstrating a 13% risk reduction compared to placebo (RR, 0.87; 95% CI, 0.75-1.01). While not quite reaching statistical significance, this trend is noteworthy. Dapagliflozin follows closely, with an 11% risk reduction versus placebo (RR, 0.89; 95% CI, 0.79-1.00). The upper bound of the confidence interval just touching 1.00 suggests a borderline significant effect. SUCRA scores (dapagliflozin, 0.66; empagliflozin, 0.81; and placebo, 0.03) further support the potential benefits of both SGLT2 inhibitors in myocardial infarction risk reduction, with empagliflozin slightly edging out dapagliflozin in this ranking.

Hospitalization for Heart Failure

**Figure 6:**
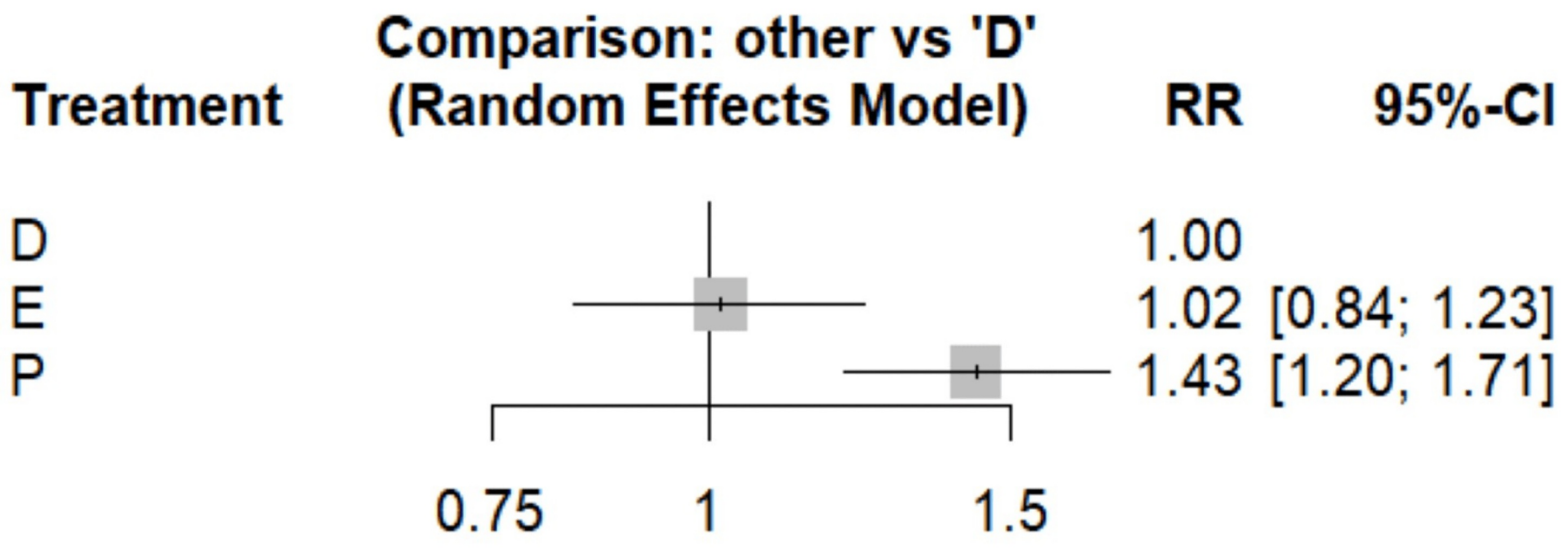
Forest plot comparing the risk of hospitalization for heart failure References: [13,15,16,19,20,22,24] D, dapagliflozin; E, empagliflozin; P, placebo; RR, risk ratio; CI, confidence interval

Figure 6 compares the risk of hospitalization for heart failure between dapagliflozin and empagliflozin. No significant difference was found between the two groups in terms of hospitalization for heart failure (RR, 1.02; 95% CI, 0.84-1.24), suggesting similar efficacy between the two medications. As shown in Table 4, both SGLT2 inhibitors demonstrate significant reductions in hospitalization for heart failure risk compared to placebo. Dapagliflozin shows a 30% risk reduction (RR, 0.70; 95% CI, 0.59-0.84), while empagliflozin exhibits a nearly identical 29% reduction (RR, 0.71; 95% CI, 0.59-0.86). These results are statistically significant, as evidenced by the confidence intervals not crossing 1. The SUCRA scores (dapagliflozin, 0.815; empagliflozin, 0.685; and placebo, 0.001) further emphasize the superiority of both SGLT2 inhibitors over placebo, with dapagliflozin slightly favored in this ranking for preventing hospitalizations for heart failure.

Discussion

Our meta-analysis compared dapagliflozin and empagliflozin to placebo for cardiovascular outcomes. The pooled analysis showed that dapagliflozin is more effective in preventing atrial fibrillation compared with empagliflozin. However, no significant differences are reported in terms of other outcomes in terms of MACE, cardiovascular mortality, hospitalization for heart failure, and myocardial infarction.

The protective effect of SGLT2 inhibitors against incident atrial fibrillation in T2D patients has been explained by a number of mechanisms. These possible causes include oxidative stress, arrhythmogenic epicardial fat, and decreases in the electrical and structural remodelling of the atrium [25,26]. However, the precise mechanism behind dapagliflozin's unique and superior advantage is still unknown. Our findings may be explained by dapagliflozin's stronger SGLT2 and SGLT1 affinity than empagliflozin, if the anti-AF mechanisms discussed above represent a class effect shared by all SGLT2 inhibitors [27]. 

This meta-analysis found no significant difference in cardiovascular outcomes when comparing empagliflozin with dapagliflozin. Prior meta-analyses of large cardiovascular outcome trials (CVOTs) have shown that both dapagliflozin and empagliflozin lead to favorable composite cardiovascular outcomes, including reductions in cardiovascular death, myocardial infarction (MI), and ischemic stroke, as well as a decrease in hospitalization for heart failure, compared to placebo in patients with T2DM [28,29]. Interestingly, our study introduces a new perspective to the existing literature by directly comparing these two leading SGLT2 inhibitors. The absence of a significant difference in cardiovascular outcomes between dapagliflozin and empagliflozin offers clinicians more flexibility in their prescribing decisions, enabling them to take into account factors such as patient preferences, cost, and potential side effects when choosing between these medications.

Additionally, hospitalization for heart failure is considerably reduced in patients on dapagliflozin or empagliflozin, according to our meta-analysis. We have discussed the potential reasons for the decrease in heart failure associated with dapagliflozin and empagliflozin. For instance, prior research showed that dapagliflozin improves cardiometabolic indicators, vascular remodelling, and left ventricular diastolic functioning [30,31]. In patients with type 2 diabetes, empagliflozin has also been linked to positive changes in left ventricular mass and diastolic function, which may reduce the risk of heart failure [32]. Although both SGLT2 inhibitors appear to lower the risk of heart failure, we believe that dapagliflozin may have a little higher effect on heart failure reduction than empagliflozin based on data from prior studies and our analyses.

Clinical Implications

This meta-analysis reinforces the cardiovascular benefits of both dapagliflozin and empagliflozin in patients with T2DM. The comparable efficacy in reducing MACE, cardiovascular mortality, myocardial infarction, and hospitalization for heart failure allows clinicians flexibility in prescribing. However, the lower atrial fibrillation risk with dapagliflozin may influence treatment decisions for patients with or at risk of AF. The significant reduction in hospitalization for heart failure underscores the importance of considering SGLT2 inhibitors in patients with T2DM and cardiovascular risk factors.

Research Implications

Future studies should focus on elucidating the mechanisms behind dapagliflozin's superior effect on atrial fibrillation risk. Direct head-to-head trials comparing dapagliflozin with empagliflozin are needed to confirm these findings. Additionally, research into the potential differences in SGLT2 and SGLT1 affinity between these drugs and their impact on cardiovascular outcomes could provide valuable insights. Long-term studies evaluating the sustained cardiovascular benefits and potential side effects of these SGLT2 inhibitors are also warranted.

Limitations

The present meta-analysis has certain limitations. Firstly, most included studies were observational in nature, and they are associated with selection bias. Secondly, atrial fibrillation was assessed by only four studies. We need studies in the future to compare the risk of atrial fibrillation between two drugs. Thirdly, we were not able to assess publication bias as the number of studies assessed for each outcome was less than 10. Lastly, we were not able to perform subgroup analysis due to the lack of individual-level data.

## Conclusions

Based on the meta-analysis results, both dapagliflozin and empagliflozin demonstrate significant cardiovascular benefits for patients with type 2 diabetes mellitus compared to placebo. These SGLT2 inhibitors show comparable efficacy in reducing major adverse cardiovascular events, cardiovascular mortality, myocardial infarction, and hospitalization for heart failure. However, dapagliflozin appears to have a slight edge in preventing atrial fibrillation. While these findings provide valuable insights for clinical decision-making, some limitations exist, including potential selection bias in observational studies and limited data on atrial fibrillation. Future research should focus on direct head-to-head trials, exploring mechanisms behind dapagliflozin's superior effect on atrial fibrillation, and conducting long-term studies to evaluate the sustained benefits and potential side effects of these SGLT2 inhibitors.

## Appendices

Table 6 shows the GRADE NMA assessment.

**Table 6:** GRADE NMA assessment Publication bias is unable to be assessed because there are less than 10 studies available MACE, major adverse cardiovascular events; GRADE, Grading of Recommendations Assessment, Development and Evaluation; NMA, network meta-analysis

Outcome	Comparison	Inconsistency	Indirectness	Imprecision	Publication bias	Certainty of evidence
MACE	Dapagliflozin and empagliflozin	Serious	Not serious	Not serious	Unclear	Moderate
	Dapagliflozin and placebo	Not serious	Not serious	Serious	Unclear	Moderate
	Empagliflozin and placebo	Not serious	Not serious	Serious	Unclear	Moderate
Atrial fibrillation	Dapagliflozin and empagliflozin	Serious	Not serious	Not serious	Unclear	Moderate
	Dapagliflozin and placebo	Serious	Not serious	Not serious	Unclear	Moderate
	Empagliflozin and placebo	Serious	Not serious	Serious	Unclear	High
Cardiovascular mortality	Dapagliflozin and empagliflozin	Not serious	Not serious	Not serious	Unclear	Low
	Dapagliflozin and placebo	Serious	Not serious	Serious	Unclear	High
	Empagliflozin and placebo	Not serious	Not serious	Serious	Unclear	Moderate
Myocardial infarction	Dapagliflozin and empagliflozin	Not serious	Not serious	Not serious	Unclear	Low
	Dapagliflozin and placebo	Not serious	Not serious	Not serious	Unclear	Low
	Empagliflozin and placebo	Not serious	Not serious	Not serious	Unclear	Low
Hospitalization for heart failure	Dapagliflozin and empagliflozin	Not serious	Not serious	Not serious	Unclear	Low
	Dapagliflozin and placebo	Serious	Not serious	Not serious	Unclear	Moderate
	Empagliflozin and placebo	Not serious	Not serious	Not serious	Unclear	Low
